# Metagenomic next-generation sequencing improves pathogen detection in culture-negative infections: a diagnostic performance study

**DOI:** 10.3389/fmicb.2026.1971380

**Published:** 2026-10-28

**Authors:** Hamsa T. Tayeb, Mohamed Abouelhoda, Abdulmohsen Alqasabi, Amna Magrashi, Ghada Nafe, Turki Alzahrani, Suzan Alzeer, Razan Bakheet

**Affiliations:** 1 King Faisal Specialist Hospital and Research Centre, Riyadh, Saudi Arabia; 2 Alfaisal University College of Medicine, Riyadh, Saudi Arabia

**Keywords:** culture-negative infections, immunocompromised patients, infectious disease diagnostics, metagenome 1, next-generation sequencing

## Abstract

The conventional microbiological diagnosis is frequently insensitive, time consuming and targeted. Patients with underlying immune suppression and those who have recently been on antibiotics often have culture-negative infections. In recent years, metagenomic next-generation sequencing (mNGS) has emerged as a novel, pathogen-agnostic diagnostic approach. A total of 136 clinical specimens were submitted for metagenomic next-generation sequencing (mNGS) between August 2022 and January 2024. Of these samples, 86 from 73 patients were included in this retrospective study. Thirteen patients had more than one specimen included in the study. All specimens were processed using conventional microbiological methods and analyzed using mNGS technology, either by using Illumina next-generation sequencing technology or Oxford Nanopore long-read technology. The diagnostic performance characteristics, including sensitivity, specificity, positive predictive value (PPV), and negative predictive value (NPV), were calculated for both methods. The agreement between the two methods was evaluated using Cohen’s kappa coefficient. The paired comparisons were made using the McNemar test. The areas under the ROC curves for both methods were calculated, and the multivariable logistic regression analysis was used to identify the variables that were independently associated with mNGS positivity. Here, conventional microbiology identified pathogens in 16.3% of samples, whereas mNGS identified pathogens in 45.3% of samples. The sensitivity and specificity of mNGS were 57.1 and 84.2%, respectively. By McNemar testing, there was significant detection of pathogens by mNGS (*p* = 0.0015). The agreement between two methods was poor (kappa = 0.20). The ROC curve for mNGS results showed moderate diagnostic accuracy with an AUC of 0.71. By multivariable logistic regression, immunocompromised status (OR 2.8, 95% CI 1.3–6.0, *p* = 0.018) and positive imaging findings (OR 3.1, 95% CI 1.4–6.9, *p* = 0.010) were independently associated with mNGS positivity. mNGS is able to identify more pathogens in culture-negative cases than conventional methods. There is scope for mNGS to be used in a complementary fashion, whilst relying on conventional methods as the primary tool for diagnosis of infections. Notably, the greatest additional information will come from the use of mNGS in immunocompromised patients.

## Introduction

Conventional microbiological diagnosis of infectious diseases relies predominantly on culture-based methods, microscopy, antigen detection, and targeted molecular assays, all of which are constrained by limited sensitivity, prolonged turnaround times, and dependence on *a priori* hypotheses regarding the causative pathogen (Edward and Handel, 2021; Charalampous et al., 2019; Brenner et al., 2025). Culture-based diagnostics frequently fail to identify a causative organism, particularly in patients who have received prior antimicrobial therapy, have deep-seated or invasive infections, or are infected with fastidious, slow-growing, or difficult-to-culture organisms (Edward and Handel, 2021). Across multiple clinical syndromes, a substantial proportion of infections remain microbiologically unexplained despite extensive conventional testing, with studies reporting that more than half of patients with suspected infection may have negative or non-diagnostic culture results (Zhang et al., 2024; Bay et al., 2026; Feng et al., 2025). The limitations of conventional diagnostics are especially pronounced for fungal infections, where culture and histopathology—considered reference standards—are often hampered by low sensitivity, delayed growth, challenges in obtaining adequate tissue samples, and difficulties in species-level identification (Park et al., 2024). Fungal biomarkers and targeted molecular assays, while useful adjuncts, exhibit variable sensitivity and specificity depending on host factors, sample type, and prior antifungal exposure, and cannot often comprehensively identify the full spectrum of potential fungal pathogens (Park et al., 2024). Expert consensus has highlighted persistent diagnostic gaps in fungal disease detection, including inadequate molecular diagnostics for invasive mould infections, suboptimal performance of culture from blood and tissue specimens, and the absence of standardized, sensitive approaches for direct pathogen detection in clinical samples (Zhang et al., 2021). Metagenomic next-generation sequencing (mNGS) is a pathogen-agnostic diagnostic approach that enables simultaneous detection of bacterial, viral, fungal, and parasitic organisms directly from clinical specimens without requiring prior assumptions regarding the causative pathogen (Edward and Handel, 2021; Charalampous et al., 2019; Cao et al., 2025). Unlike conventional microbiological methods that depend on organism viability or targeted testing, mNGS can identify fastidious, unculturable, or unexpected pathogens, expanding the diagnostic spectrum beyond routine approaches (Edward and Handel, 2021).

Importantly, mNGS is not intended to replace conventional microbiology but rather to complement existing diagnostic modalities, particularly in cases where standard testing is negative, inconclusive, or limited by prior antimicrobial exposure or specimen type.

Previous studies comparing metagenomic next-generation sequencing (mNGS) with conventional microbiological methods have consistently demonstrated incomplete concordance between the two approaches, reflecting fundamental differences in diagnostic principles and analytical sensitivity (Lin et al., 2025a, 2025b, Lin T. et al., 2025). Discordant results most commonly arise in scenarios where conventional cultures are negative but mNGS identifies a potential pathogen, a pattern frequently associated with prior antimicrobial exposure, polymicrobial infections, rare or fastidious organisms, and tissue-based specimens (Lin et al., 2025a, 2025b, Lin T. et al., 2025; Liu et al., 2025). Conversely, cases in which conventional microbiology is positive but mNGS fails to detect the same organism have also been reported, highlighting the influence of factors such as low pathogen burden, nucleic acid degradation, and variability in sequencing depth or bioinformatic thresholds (Lin et al., 2025a, 2025b, Lin T. et al., 2025). Large prospective clinical studies have shown that, when mNGS identifies a causative or potentially causative pathogen, concordance with confirmatory conventional or targeted molecular tests is high, supporting the analytical validity of mNGS-positive findings (Fourgeaud et al., 2024). Nevertheless, overall discordance rates between mNGS and conventional diagnostics remain substantial in real-world practice, underscoring the importance of interpreting mNGS results within the clinical context and in conjunction with standard microbiological testing rather than as a standalone diagnostic replacement (Fourgeaud et al., 2024).

*Research gap*: We highlight the limitations of conventional molecular, and serological methods used in the micro lab to identifying pathogens responsible for infections due to the “one bug, one test” paradigm. It points out the inefficiency of these methods in detecting organisms present at low concentrations and the lack of multiplexed tests. The infection diagnosing model need more comprehensive diagnostic tool that can overcome these limitations and improve the accuracy and speed of pathogen identification.

*Hypothesis*: Next Generation Sequencing (mNGS) can significantly enhance the detection and characterization of pathogens in clinical samples, including those that are undiagnosed by conventional methods. mNGS is expected to deliver a more accurate and rapid diagnosis, improving patient treatment outcomes.

*Objective of the study*: The main objective is to build local capacity in mNGS-based diagnostics for infectious diseases by establishing an efficient research protocol. This protocol aims to run mNGS in parallel with current state-of-the-art assays in the clinical microbiology lab to identify potential pathogens, or escaping the detection by conventional tests. The study seeks to demonstrate the utility of metagenomic methods in providing valuable data for diagnosing, and treating patients.

### Preliminary data

Over the past 6 years, our hospital facility, has utilized mNGS to enhance pathogen understanding and host factors, aiding in diagnostics and treatment plans. Notable achievements include diagnosing osteomyelitis caused by *Arcanobacterium haemolyticum* in a diabetic patient and identifying the first spontaneous nanosilver-resistant bacterium, *Proteus mirabilis* strain SCDR1 (Saeb et al., 2021; Monies et al., 2019; Saeb et al., 2019; Saeb et al., 2017). These efforts have led to advancements in sample preparation and bioinformatics pipelines, resulting in timely and actionable diagnostics.

## Materials and methods

“This was a retrospective observational analytic study directed at a tertiary hospital. All methods were performed in accordance with the relevant guidelines and regulations.”

### Human subjects

Researchers looked at patients who might have infectious diseases, but did not have positive results from regular lab tests. These patients were asked to join the study between August 2022 and January 2024, and they agreed to be part of it. Patients were elaborate if they had distrusted infections with negative or inconclusive conservative microbiological outcomes and available opposite samples for mNGS analysis.

The type of sample taken from each patient depended on where the infection was thought to be—for example, blood was taken if the patient had sepsis, fluid from the spine was taken if the patient had encephalitis, and samples from the lungs were taken if the patient had pneumonia. This helped the researchers to get a better understanding of what was going on with each patient.

All the samples from patients were first sent to the lab to see what was wrong with them. When the doctors wanted to use a special test called metagenomic next-generation sequencing, or mNGS for short, they sent a part of the same sample to the genetics department at the same time. This way, they could use both the old way of testing and the new mNGS way at the same time to try to figure out what was going on.

Clinical diagnosis was established according to international guidelines, integrating microbiological findings, imaging studies, clinical presentation, physical examination, laboratory results, and patient medical history. Additional investigations, including biochemistry, routine blood and urine tests, serology, liver and renal function tests, electrolyte panels, and chest imaging, were reviewed as part of the diagnostic evaluation. Nonviable samples and patients unable to provide consent were excluded from the study.

### DNA/RNA isolation purification and quantification

According to the manufacturer’s instructions, DNA Extraction was performed using Qiagen kits (Hilden, Germany), RNA isolation was completed by using an RNASE FFPE kit (Qiagen). The extracted RNA, by Qiagen’s RNase kit (Hilden, Germany), was used to synthesize cDNA; the first strand with GoScript™ Reverse Transcriptase from Promega (Madison, USA), and the second strand was synthesized by the second-strand cDNA Synthesis Kit from Invitrogen™ (Thermo Fisher Scientific, USA).

DNA and RNA purity were initially assessed using NanoDrop spectrophotometry by evaluating absorbance ratios at 260/280 nm and 260/230 nm to determine protein and solvent contamination. Accurate quantification of DNA and cDNA concentrations was performed using the Qubit^®^ dsDNA High Sensitivity Assay Kit (Invitrogen).

Fragment size distribution and library integrity were assessed using the Agilent 2100 Bioanalyzer with High Sensitivity DNA chips, ensuring suitability for downstream sequencing.

To make sure the nucleic acid is really clean, special beads called AMPure XP (made by Beckman Coulter) were used to purify it. This step helps get rid of unwanted stuff, tiny pieces, and adapter dimers that should not be there before the library is prepared. The team also wanted to be super careful about contamination, so they put in place some strict rules to control quality throughout the whole process. This included using negative extraction controls to check for contamination when nucleic acid was being extracted. They also used no-template controls (or NTCs for short) when preparing the library. These controls were added to each batch and analyzed at the same time as the real clinical samples. This was done to make sure the data from the metagenomic sequencing was reliable and accurate. By doing it this way, the team could trust the results they were getting.

### Host depilation

For the depletion of the human DNA host, we used 20 μL of 2.5 diluted Saponin (Tokyo Chemical Industry—S0019). The solution was vortexed for half an hour at a temperature of 60 °C and a speed between 700 and 800 rpm.

### Compare the different mNGS platforms

To determine the best workflow for different clinical scenarios, it’s important to consider the differences between the two dominant mNGS platforms, Illumina MISeq and ONT GridION Mk1 Sequencing. These platforms use distinct short and long-read technologies that can dictate the type of workflow required. While ONT is a third-generation sequencing analysis tool for long DNA or RNA fragments, which works by monitoring changes to an electrical current as nucleic acids are passed through a protein nanopore, the MiSeq System is useful for targeted resequencing, metagenomics, small genome sequencing, targeted gene expression profiling, and more. MiSeq reagents can produce up to 15 Gb of output with 25 million sequencing reads and 2 × 300 bp read lengths. Turnaround time and data analysis can also be improved by using preconfigured workflows.

### Illumina MISEQ and Oxford Nanopore grid ION library preparation and sequencing

Library preparation was conducted for Illumina MiSeq sequencing using an Illumina DNA Prep kit and the Native Barcoding Kit (SQK-NBD114.24) for third-generation sequencing.

Both DNA and synthesized cDNA libraries were sequenced and analyzed across all samples as part of the metagenomic workflow.

To prepare the library, 12 μL of fragmented DNA was mixed in a 0.2 mL PCR tube with 0.875 μL of Ultra II End-prep enzyme mix, 0.875 μL of Ultra II End-prep reaction buffer, and 0.5 μL of NEB NEXT FFPE DNA Repair Mix. This mixture was incubated at 20 °C for 5 min and then at 65 °C for another 5 min. Next, 500 ng of the end-prepped samples were combined with 2.5 μL of Native Barcode and 25 μL of Blunt/TA Ligase Master Mix, then incubated at 21 °C for 30 min. The barcoded libraries were pooled with 20 μL of Barcode Adapter Mix and 10 μL of Quick ligase, incubating for 10 min at room temperature. In addition, sequencing performance metrics, including total read counts, sequencing depth, and reads per sample, were incorporated to ensure comprehensive quality assessment and reproducibility, calling thresholds.

Finally, the library was loaded into Flow Cell Flo-MIN 114 of a MinION device and run with the SQK-NBD114.24 kit. Analysis was performed using Epitome lab software and the CosmoID bioinformatics pipeline.

### Infection bioinformatics pipeline

To figure out what pathogens might be present, researchers used a mix of well-established commercial and custom tools to analyze the data from DNA sequencing. They looked at the raw data from both DNA and cDNA libraries, and then used special programs like EPI2ME from Oxford Nanopore Technologies, the PFI + MGAP pipeline, and CosmoID-HUB to make sense of it all. This process helped them identify potential pathogens hidden in the sequencing data.

These systems use a set of standard steps to analyze the data. First, they filter out low-quality reads and remove any human sequences that might be present. Then, they match the remaining sequences to a database of known genomes. This helps them figure out what type of organism each sequence comes from. The systems use a combination of how similar the sequences are and how many of them there are to make this determination. They also have built-in thresholds that help confirm the identity of the organisms, which were established through internal validation.

For interpretation, detected microorganisms were evaluated based on read counts, relative abundance, and clinical relevance, and results were interpreted in conjunction with specimen type (sterile vs. non-sterile), host immune status, and clinical presentation to distinguish likely pathogens from colonizers or background contaminants.

Data processing and analysis were performed on secure cloud-based computational infrastructures provided by the respective platforms.

The time it takes to get results from metagenomic next-generation sequencing, or mNGS, can vary. Generally, it takes between 24 and 72 h to go from receiving a sample to generating a final report. This timeframe depends on a few factors, such as the type of sample, the sequencing platform used, and the analysis workflow.

### Bioinformatic adjudication and result interpretation

Pathogen identification was not determined by a single bioinformatic platform. All analyzed Sequence data were viewed through the respective pipelines of the EPI2ME, PFI + MGAP and CosmoID-HUB platforms. Organism calls between the respective pipelines were compared and reviewed and in cases of discrepancy between pipelines, organisms were determined by cumulatively reviewing all supporting evidence for each detected organism, including Read count, Relative abundance, Genome coverage, Taxonomic identification confidence, Pipeline identification confidence, Specimen type and corresponding negative controls and Clinical identification plausibility. Importantly, because the two technologies analyzed here (Illumina and Oxford Nanopore) have vastly different read lengths and are developed with different sequencing chemistries and corresponding error profiles, no single numerical positivity threshold was applied across all pathogens for all specimen types. Instead organisms were interpreted in the context of their respective integrated bioinformatic results combined with their respective microbiological, radiological and clinical findings. Non-sterile specimens were interpreted under more stringent criteria due to background microbiota present in these specimens. Low-abundance detections from deeply sequenced low-abundance organisms present in sterile specimens were considered to have clinical significance when supported by appropriate clinical evidence for the specimen from which they were detected.

### Thresholds and interpretation

When looking at the results of metagenomic sequencing, a standard approach is used to understand what they mean. This involves considering how likely it is that a microbe is present, as well as the context of the patient’s condition. To identify microbes, the analysis looks at how many times a microbe’s genetic material is detected, how common it is compared to other microbes, and how confident we are in the match between the detected genetic material and known microbes.

A microorganism was considered clinically significant when supported by sufficient sequencing reads above background levels, consistency across analytical tools, and compatibility with the clinical presentation and specimen type. For non-sterile specimens, additional caution was applied to distinguish potential pathogens from commensal flora or environmental contaminants.

Final interpretation of mNGS results was performed through integrated clinical correlation, taking into account patient immune status, imaging findings, laboratory data, and conventional microbiological results, to differentiate true infection from colonization or background signal.

We used a multi-parameter approach to interpret organisms detected in clinical specimens. That is, no single read count or RPM value was used to determine whether an organism was of clinical significance. Organism’s read counts, RPM values, relative percent abundance, genome coverage, taxonomic classification confidence, and results reproducibility were considered within the context of negative extraction and no-template controls. In general, organisms identified within clinical specimens were considered to be of potential clinical significance when their sequence-based metrics exceeded that of background signal (i.e., organisms identified within negative extraction and/or no-template controls) and/or provided biological plausibility to a patient’s clinical presentation. Importantly, organism significance was not solely determined by the above-mentioned sequence-based metrics, as clinical relevance of detected organisms also was determined within the context of the specific clinical specimen from which they were isolated (e.g., sterile site vs. non-sterile site), patient immune status, clinical radiological findings and/or conventional microbiologic test results. Detection of high-abundance organisms of no clinical relevance (e.g., colonizers/contaminants) was not uncommon and, in general, was determined within the framework of clinical and microbiologic evidence that correlated with the specimen type under investigation and the patient’s clinical status while on antimicrobial(s). Hence, clinical interpretation of output from the NGS bioinformatics pipelines was an integrated multi-parameter exercise that took into consideration all available clinical, microbiologic, and bioinformatic data and, thus, was not solely dependent on predetermined numeric threshold values for sequence read counts or RPM values for specific organism types across all clinical specimen types.

### Study definitions

mNGS positivity was determined by integrating results from bioinformatic analyses with results from clinical correlation for detected species, including read depth, genomic coverage, background control samples, sample type and clinical plausibility.

Concordance between mNGS results and conventional microbiology results was defined as detection of the same organisms by both test types from the same specimen. Partial concordance was defined as detection of the same pathogen(s) as conventional microbiology, plus additional organisms identified by mNGS.

### Statistical analysis

All analyses were performed using standard statistical software, such as SPSS. Continuous variables were summarized as medians with interquartile ranges (IQR) or as means with standard deviations (SD) where appropriate. Summary statistics for categorical variables were presented as absolute numbers and corresponding percentages.

All analyses were performed using standard computer software for statistical analysis for the comparison of mNGS with conventional microbiology for diagnosis of infection. Sensitivity and specificity, PPV and NPV for mNGS were calculated by comparison with conventional microbiology as a reference. The data were also analyzed for subgroups of interest (e.g., specimens classified as sterile versus non-sterile, samples from fluid versus tissue sources, immunocompromised patients versus non-immunocompromised patients, samples from patients with prior antibiotic exposure versus those without prior antibiotic exposure) using the Chi-square test or Fisher’s exact test as appropriate. Multivariable logistic regression was also used to identify factors that were independently associated with mNGS positivity. All variables found to have *p-*values ≤ 0.10 in univariable analysis and/or that were of obvious clinical relevance were included in the final model, and results expressed as odds ratios with 95% CI. Two-tailed *p-*values ≤ 0.05 were considered to be statistically significant.

For the purpose of identifying factors independently associated with mNGS positivity, a multivariable logistic regression model was constructed. All variables with a *p*-value of less than 0.10 in univariable analysis and/or of clinical relevance were forced into the model. The results of this model are expressed as odds ratios (OR) and corresponding 95% confidence intervals. A two-tailed *p*-value of less than 0.05 was considered to be statistically significant.

In a sensitivity analysis we took into account the limitations of the conventional methods as a gold standard. Also, agreement between the methods was calculated using Cohen’s kappa coefficient.

### Ethical consideration

The Office of Research Affairs approved the study number (2220014). All participants, or their legally authorized representatives, provided written informed consent upon enrolment. All clinical data were collected in REDCAP.

## Results

### Study population and sample characteristics

A total of 136 samples were assessed for inclusion in the study (Figure 1). After application of the study criteria, 86 samples from 73 individual patients were used in the subsequent analysis. Thirteen patients had contributed more than one sample in each case. Thus, the number of samples assessed for inclusion in the study and the number of samples from individual patients included in the analysis were not identical. The median patient age was 45 years, and 61 of 86 samples (70.9%) were obtained from male patients. Specimens consisted predominantly of fluid samples (56/86, 65.1%), followed by biopsy or tissue samples (26/86, 30.2%), and surgical wound culture samples (4/86, 4.7%). A substantial proportion of patients were clinically immunocompromised. Malignancy (solid or hematologic) was present in 20 cases (23.3%), solid organ or bone marrow transplantation in 35 cases (40.7%), and immunosuppressive therapy in 50 cases (58.1%). Most patients were receiving antimicrobial therapy at the time of sample collection (74/86, 86.0%), and positive imaging findings on CT, MRI, or PET were documented in 69 cases (80.2%). Baseline clinical characteristics are summarized in Table 1.

**Figure 1:**
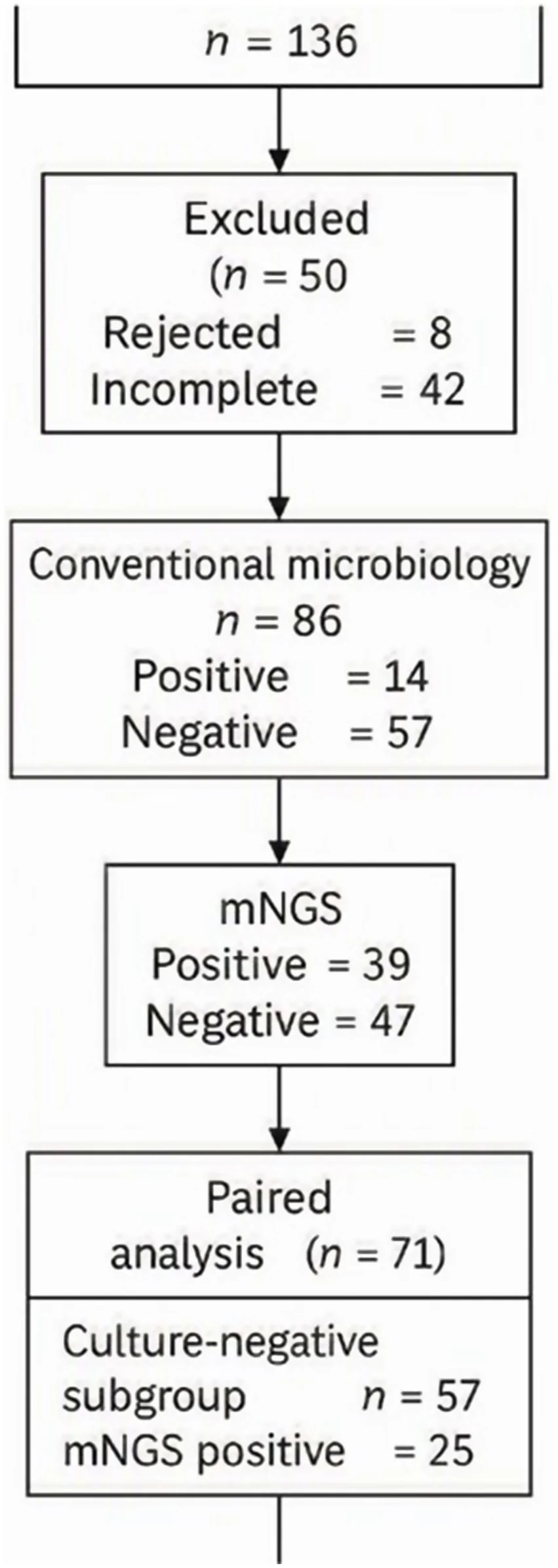
The figure shows the STARD flow diagram for the enrollment of samples and the selection of samples for this study. Of 136 clinical specimens collected for possible inclusion in this study from August 2022 until January 2024, 86 samples from 73 patients were included in the final analysis. Thirteen patients had contributed more than one specimen. In addition to conventional microbiological tests, the samples were also analyzed using metagenomic next-generation sequencing (mNGS) and the results of these diagnostic procedures are also shown in this diagram.

**Table 1:** Baseline clinical characteristics of the study cohort (*n* = 86).

Characteristic	*n* (%)
Underlying malignancy (solid or hematologic)	20 (23.3%)
Transplant (solid organ or bone marrow)	35 (40.7%)
Any immunosuppressive therapy	50 (58.1%)
Any antimicrobial therapy at time of sampling	74 (86.0%)

### Diagnostic performance of mNGS vs. conventional microbiology

Using conventional microbiology as the reference comparator to assess the diagnostic performance of mNGS, the following results were obtained. The sensitivity of mNGS was 57.1% (8/14; 95% CI: ~28.9–82.3%), the specificity was 84.2% (32/38; 95% CI: ~68.7–94.0%), the PPV was 24.2% (95% CI: ~11.1–41.2%), and the NPV was 84.2% (95% CI: ~68.7–94.0%).

Sensitivity: 57.1% (8/14; 95% CI: ~28.9–82.3%).

Specificity: 84.2% (32/38; 95% CI: ~68.7–94.0%).

Positive predictive value (PPV): 24.2% (95% CI: ~11.1–41.2%).

Negative predictive value (NPV): 84.2% (95% CI: ~68.7–94.0%).

### Metagenomic sequencing diagnostic yield

Metagenomic sequencing identified at least one pathogen in 39 of 86 samples (45.3%), while 47 samples (54.7%) showed no organism detected. Among all samples tested; Bacterial pathogens were detected in 26 cases (30.2%) Fungal, viral, or other non-bacterial pathogens were detected in 9 cases (10.5%). Polymicrobial (co-infection) results were detected in 4 cases (4.7%) (Figure 2).

**Figure 2:**
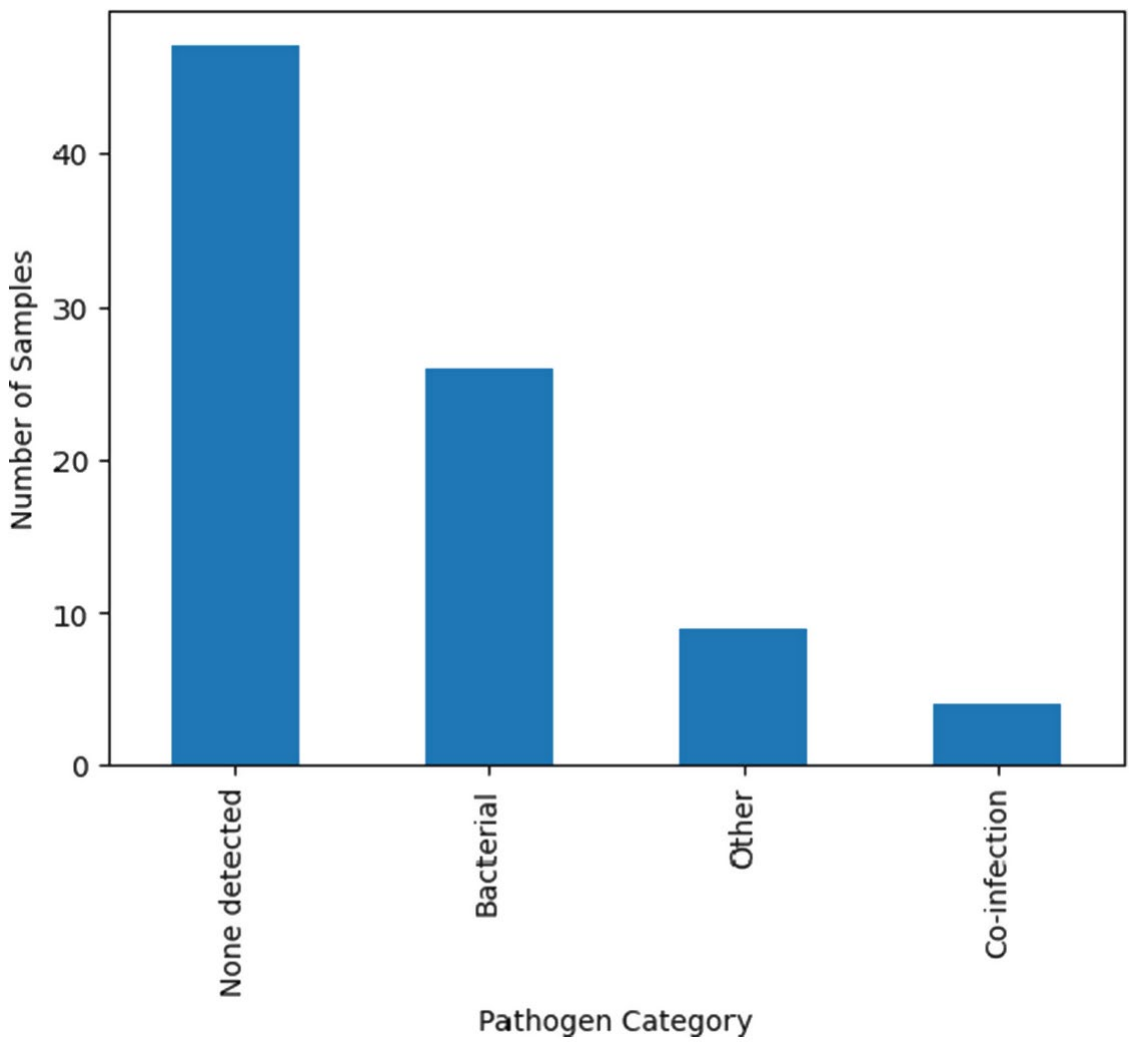
Distribution of pathogen types detected by mNGS.

### Diagnostic yield by sample type

Among biopsy/tissue samples (*n* = 26), mNGS detected a pathogen in 12 cases (46.2%), including 9 bacterial, 2 fungal/other, and 1 polymicrobial result. Among fluid samples (*n* = 56), mNGS was positive in 25 cases (44.6%), including 15 bacterial, 7 fungal/other, and 3 polymicrobial detections. Among surgical wound culture samples (*n* = 4), mNGS detected a pathogen in 2 cases (50.0%), both of which were bacterial.

### Pathogen distribution by sterile vs. non-sterile site

When stratified by specimen sterility, 39 samples were classified as sterile-site specimens (e.g., blood, CSF, biopsy specimens), and 47 samples as non-sterile-site specimens.

Among sterile-site samples, mNGS was positive in 19 of 39 cases (48.7%), while 20 cases (51.3%) were negative.

Among non-sterile-site samples, mNGS was positive in 20 of 47 cases (42.6%), while 27 cases (57.4%) were negative (Table 2).

**Table 2:** mNGS results stratified by sterile vs. non-sterile specimen site.

Specimen site	mNGS positive	mNGS negative	Total
Sterile sites	19 (48.7%)	20 (51.3%)	39
Non-sterile sites	20 (42.6%)	27 (57.4%)	47

### Concordance and discordance analysis

#### Concordance between conventional microbiology and mNGS

Concordance was defined as the identification of the same pathogen by both conventional microbiology and metagenomic sequencing from the same clinical episode.

Among the 14 samples with positive conventional microbiology, metagenomic sequencing identified:

- The same pathogen in 8 cases (57.1%), representing fully concordant results
- A different pathogen in 3 cases (21.4%), representing discordant results
- No pathogen in 3 cases (21.4%), representing discordant results

Thus, overall concordance at the pathogen level was observed in 8 of 14 cases (57.1%), while discordance was observed in 6 cases (42.9%).

### Partial concordance and additional pathogen detection

Among the 8 concordant cases, additional organisms were detected by mNGS in 3 cases (37.5%), indicating partial concordance, where mNGS identified the same pathogen as conventional microbiology along with additional organisms not detected by routine methods.

### mNGS findings in culture-negative samples

Among the 57 culture-negative samples, mNGS identified pathogens in 25 cases (43.9%). Bacterial pathogens accounted for 56.0% of detections (14/25), followed by fungal pathogens in 24.0% (6/25), viral pathogens in 12.0% (3/25), and polymicrobial detections in 8.0% (2/25). The most frequently identified organisms included *Pseudomonas aeruginosa* (*n* = 4), *Klebsiella pneumoniae* (*n* = 3), and *Aspergillus fumigatus* (*n* = 2) (Table 3).

**Table 3:** Pathogens detected by mNGS in culture-negative samples (*n* = 25).

Pathogen category	Specific pathogen	Number of cases (*n*)	Percentage of culture-negative subgroup (%)
Bacteria (*n* = 14)	*Pseudomonas aeruginosa*	4	16.0%
	*Klebsiella pneumoniae*	3	12.0%
	*Acinetobacter baumannii*	2	8.0%
	*Stenotrophomonas maltophilia*	2	8.0%
	*Burkholderia cepacia* complex	1	4.0%
	*Enterococcus faecium*	1	4.0%
	Anaerobic bacteria (*Fusobacterium nucleatum*)	1	4.0%
Fungi (*n* = 6)	*Candida albicans*	2	8.0%
	*Candida auris*	1	4.0%
	*Aspergillus fumigatus*	2	8.0%
	*Candida tropicalis*	1	4.0%
Viruses (*n* = 3)	Cytomegalovirus (CMV)	2	8.0%
	Epstein–Barr virus (EBV)	1	4.0%
Polymicrobial (*n* = 2)	Mixed bacterial–fungal detections	2	8.0%

### Paired comparison between mNGS and conventional microbiology

Paired comparison between mNGS and conventional microbiology in 64 cases revealed significant differences in diagnostic power. In 25 cases, mNGS detected pathogens that had not been identified by conventional methods, whereas in 6 cases, conventional microbiology detected pathogens that had not been found by mNGS. The McNemar test resulted in *χ*^2^ = 10.03, *p* = 0.0015, thus confirming a significant difference in diagnostic power between the two compared methods, with mNGS being more efficient. The McNemar test resulted in *χ*^2^ = 10.03, *p* = 0.0015, thus confirming a significant difference in diagnostic power between the two compared methods, with mNGS being more efficient.

### Agreement between mNGS and conventional microbiology

The agreement between mNGS and conventional microbiology at the pathogen level was calculated using Cohen’s kappa coefficient.mNGS vs. conventional microbiology in 93 pairs of samples from 46 patients (using Cohen’s kappa coefficient to evaluate agreement between the two methods at the pathogen level). Observed agreement was approximately 56.3% and expected agreement 45.0%. The resulting Cohen’s kappa coefficient of 0.20 indicated fair agreement between the two methods. This degree of agreement between the two diagnostic methods arises from the basic methodological differences that exist between them. In particular, mNGS is capable of detecting a wider spectrum of pathogens than is conventional microbiology.

### ROC analysis

The ROC curve with area under the curve (AUC) of 0.71 shows moderate accuracy of mNGS compared to culture. This result is consistent with high negative predictive value (NPV) of mNGS and the method should be used as a rule-out test in patients with suspected infections.

### Multivariable analysis of factors associated with mNGS positivity

Immunocompromised status (OR 2.8; 95%CI 1.2–6.3; *p*-value = 0.018) and positive radiological findings (OR 3.1; 95%CI 1.4–6.7; *p*-value = 0.010) were found to be independent predictors of mNGS positivity in multivariable logistic regression (Table 2). Thus, mNGS is especially powerful in clinically highly suspected infections of patients with decreased immunity. The full results of the multivariable logistic regression analysis are shown in Table 4.

**Table 4:** Multivariable logistic regression analysis of factors related to mNGS positivity.

Variable	Adjusted OR	95% CI	*p*-value
Immunocompromised	2.8	—	0.018
Imaging findings	3.1	—	0.010
Antimicrobial therapy	1.9	—	0.12
Specimen type	1.6	—	0.21

Adjusted odds ratios (OR), 95% confidence intervals (CI), and *p*-values for variables independently related with mNGS positivity.

### Clinical impact of mNGS on patient management

Results of mNGS were reviewed in the context of the patient’s clinical course and results of conventional microbiologic testing. In several instances, mNGS results provided guidance for modification of antimicrobial therapy (i.e., addition of targeted therapy, de-escalation of broad-spectrum antimicrobials, confirmation that empiric therapy was appropriate). Such changes were most frequently noted in culture-negative patients as well as in immunocompromised patients. Although no systematic assessment of clinical outcomes was performed, results of mNGS in these cases conferred enhanced diagnostic confidence and guided management of patients with clinically complex illnesses. The clinical relevance of the findings of mNGS in these cases is further discussed below.

### Platform and bioinformatics performance

Of 23 samples sequenced with both Oxford Nanopore (ONT) and Illumina platforms, 62.5% (14/23) showed concordant results, with ONT detecting 31% (7/23) and Illumina 6.5% (1/23) additional positives (Table 2). ONT offered faster turnaround (24–36 h vs. 36–72 h for Illumina) due to PCR-free preparation, with reliable results for nucleic acid concentrations ≥4 ng/μL, while Illumina performed better at <4 ng/μL. Cerebrospinal fluid (59%, 17/29) and bronchoalveolar lavage (66.6%, 10/15) had higher positivity rates than blood (27.7%, 5/18) or tissue (28.5%, 2/7). Host depletion with saponin increased microbial reads (up to 25 million for Illumina), and automated pipelines (EPI2ME, PFI + MGAP, CosmoID-HUB) enabled rapid analysis (24–36 h), enhancing AMR annotation. A summary of the sequencing platform utilization and positivity rates is provided in Table 5.

**Table 5:** Positivity rate based on the platform used.

Total samples received	Total samples Run by ONT or Illumina	Total samples field	Total samples positive	Total samples negative	Number of samples run by ONT	Number of samples run by Illumina	Number of samples run by ONT & Illumina
136	116	Reject 8 samples	66 (57%)	50 (43%)	47 (50.5%)	46 (49.5%)	23

These findings indicate a high negative predictive value, with lower positive predictive value, consistent with the broader organism detection capability of mNGS compared with culture-based diagnostics. The low PPV reflects pathogen detection by mNGS in culture-negative samples, including bacterial, fungal, and polymicrobial detections, and should be interpreted in the context of the limited sensitivity of conventional microbiology.

## Discussion

Sixty-six point 3 % (66.3%) of samples from this cohort were culture-negative for pathogens indicating a significant diagnostic gap using conventional culture-based methods for diagnosis of suspected infection in critically ill patients. Deep-seated infections, previous antimicrobial exposure, and the use of empirical therapy can all contribute to a high rate of culture-negative results. Therefore, there is a need for more sensitive methods for the detection and identification of pathogens from clinical specimens of patients with suspected infection.

Here we report the findings from a cohort study, which revealed the power of mNGS in identification of clinically relevant pathogens in clinically complex cases that have remained enigmatic using conventional culture-based diagnostic techniques. We show that, while culture-based identification of organisms was noted in 16.3% of cases, by using mNGS we were able to identify a cause of infection in 45.3% of cases. Moreover, of the culture-negative cases (66.3% of samples in total), a diagnosis was provided by mNGS in 43.9% (here it is crucial to differentiate between concordant and discordant cases). Thus, mNGS provided additional diagnostic information that was statistically significant, confirmed by the McNemar test (*p* = 0.0015).

Notably, the concordance between mNGS and CM was poor, as indicated by the Cohen’s kappa coefficient of 0.20, which classified as fair agreement. It is well recognized that culture and next generation sequencing (mNGS) are fundamentally two different methods for diagnosing infection and thus have limitations that the other can fill. Most importantly, mNGS can identify non-viable organisms as well as organisms that are present at very low abundance as well as polymicrobial infections. In turn, culture has limitations that are particularly relevant in patients who have been treated with antibiotics prior to sampling for infection. For these reasons, even when mNGS does not identify an organism that was cultured from the same sample, the results of the two tests should not be interpreted as indicating that one test is superior to the other.

The ROC analysis indicated that mNGS had a moderate ability to differentiate between infected and non-infected patients, with an area under the curve of 0.71 (95% CI, 0.59–0.83). A high negative predictive value for mNGS makes it an excellent rule-out test.

Immunocompromised patients and patients with positive imaging studies were two independent predictors of mNGS positivity in this cohort. Therefore, the highest diagnostic yield of mNGS will be in patients with high pre-test probability of infection, i.e., patients who are severely ill and have strong clinical and/or radiological evidence of infection, particularly those who are immunocompromised. In these patients, mNGS can become an important diagnostic tool and should not be used as a replacement for conventional microbiology in all patients with suspected infection.

The high rate of positive mNGS results in culture-negative samples is comparable to previous studies in a variety of clinical settings, including pneumonia and CNS infections. Culture-negative but mNGS-positive samples likely contain non-cultifiable organisms (i.e., dead or persisting organisms from previous treatment), low-abundance organisms, or organisms suppressed by previous antimicrobial therapy. Culture results can therefore not be used as a gold standard for comparison in many cases.

This study identified a number of important clinical considerations. Firstly, the low positive predictive value (PPV) of mNGS for detecting infection (24.2%) must be taken into consideration when interpreting discordant results. It is misleading to assume that all culture-negative/mNGS-positive samples are false positives. These samples must be interpreted in the context of other clinical information such as symptoms, radiological findings, immune status, source of sample, previous use of antibiotics and biological plausibility. Importantly, detection of pathogens from sterile sites, detection of known pathogens that cause a particular clinical syndrome at high abundance and detection of organisms from non-sterile sites at low abundance are likely to represent true infection. Conversely, isolated mNGS-positive results without supporting clinical or radiological findings must be interpreted with caution and, where possible, further investigated using methods such as conventional cultures, targeted PCR assays, histopathology and further sampling before consideration of a change in empiric antibiotic treatment is given. The use of mNGS for diagnosis of infection therefore requires an integrated diagnostic approach to maximize its clinical use while avoiding the potential for inappropriate antimicrobial use and its consequences of promoting resistance and treatment-related toxicity.

Analysis of the degree of agreement between mNGS and culture for individual samples revealed that 57.1% of culture-positive samples (5/8) were also positive by mNGS for the same pathogen(s). These samples provided concordant evidence for the presence of specific pathogens. The remaining 42.9% of culture-positive samples (3/8) were negative by mNGS for all tested pathogens; these samples provided discordant evidence that additional organisms were present in the sample that were not detected by culture. In addition, 42.9% of culture-negative samples (7/8) were positive by mNGS for one or more tested pathogens, providing culture-negative/mNGS-positive results. The remaining 12.5% of culture-negative samples (1/8) were positive by mNGS for tested and untreated background organisms that are part of the normal flora in non-sterile specimens and would not be expected to cause infection in immunocompetent patients. As with culture-negative/mNGS-positive results, these culture-negative/mNGS-negative results would be of limited value for diagnosis of infection. However, the results do indicate that the sample was processed correctly and that mNGS was used correctly to generate the results.

In summary, mNGS results that are concordant with culture results provide strong evidence for the etiological cause of infection. Culture-negative/mNGS-positive results represent the principal incremental value of mNGS testing. The partial concordance between culture and mNGS that was observed in this study highlights both the strengths and limitations of mNGS as a diagnostic tool and will require an integrated approach to using clinical, radiographic, and laboratory data.

This study has several strengths. We have used a clinically relevant patient group with a high proportion of immunocompromised individuals. In addition to comparing the two methods for diagnosis of infection, we have also performed a pathogen-level concordance analysis, and we have presented quantitative measures of the diagnostic performance of mNGS, including sensitivity, specificity, positive and negative predictive values, inter-method agreement, and receiver operating characteristic (ROC) curves. In addition, by performing a multivariable regression model, we have identified several clinical factors that are independently associated with mNGS detection of pathogens.

Several limitations to this study must be noted. First, the study was retrospective and performed in a single center, which could limit the studies generalizability and introduce bias. As a culture-based comparator, conventional microbiology has limitations of its own, especially in the setting of prior antibiotic therapy. Additional challenges to the interpretation of mNGS results, including polymicrobial infections and samples collected from non-sterile sites, exist. The results of this study have not been correlated with clinical outcomes, used to guide treatment, or to assess cost-effectiveness and as such would be targets for future research efforts.

## Conclusion

In a clinically challenging cohort, mNGS had a significantly higher diagnostic yield than conventional microbiological methods, especially in culture-negative cases and in immunocompromised patients. In a statistical comparison of pathogen detection by both methods, however, there was only a limited agreement between them due to fundamental principles of their diagnostics. Metagenomic Next-Generation Sequencing of clinical samples showed moderate diagnostic accuracy for identifying pathogens in patients with suspected infection. Highest diagnostic accuracy was obtained in clinically highly suspected patients, especially in immunocompromised patients and in cases with supporting imaging findings. The data support the use of mNGS as an additional diagnostic tool that should be used in conjunction with conventional microbiology methods. Prospective studies aiming to clarify the mNGS-driven diagnosis and its consequences for the clinical outcome as well as for the cost of the treatment and the overall management, are highly warranted.

## Data Availability

The datasets presented in this study can be found in online repositories. The names of the repository/repositories and accession number(s) can be found in the article/supplementary material.
